# Rapid detection of grass carp reovirus type 1 using RPA-based test strips combined with CRISPR Cas13a system

**DOI:** 10.3389/fmicb.2023.1296038

**Published:** 2023-11-03

**Authors:** Huaming Li, Xinyue Cao, Ruige Chen, Min Guang, Mengran Xu, Xiaomin Wu, Rongrong Yang, Liancheng Lei, Fuxian Zhang

**Affiliations:** ^1^College of Animal Science, Yangtze University, Jingzhou, China; ^2^College of Veterinary Medicine, Jilin University, Changchun, China

**Keywords:** GCRV, CRISPR/Cas13a, RPA, detection, test strips

## Abstract

**Introduction:**

Due to the existence of grass carp reovirus (GCRV), grass carp hemorrhagic disease occurs frequently, and its high pathogenicity and infectivity are great challenges to the aquaculture industry. As a highly pathogenic pathogen, the outbreak of hemorrhagic disease often causes tremendous economic losses. Therefore, it is important to rapidly and accurately detect GCRV on site to control timely.

**Methods:**

In this study, recombinant enzyme amplification (RPA) combined with clustered regularly interspaced short palindromic repeats (CRISPR)/Cas13a system was employed to establish a method to detect the *vp7* gene of grass carp reovirus type 1. This method can be adopted for judging the results by collecting fluorescence signal, ultraviolet excitation visual fluorescence and test strip.

**Results:**

Combined with the RPA amplification experiment, the detection limit of the RPA-CRISPR method can reach 7.2 × 10^1^ copies/μL of *vp7* gene per reaction, and the detection process can be completed within 1 h. In addition, this method had no cross-reaction with the other 11 common aquatic pathogens. Then, the performance of the RPA-CRISPR/Cas13a detection method was evaluated by comparing it with the real-time fluorescence quantitative PCR detection method of clinical samples. The results of RPA-CRISPR/Cas13a detection were shown to be in consistence with the results obtained from the real-time fluorescence quantitative PCR detection. The coincidence rate of this method with 26 GCRV clinical samples was 92.31%.

**Discussion:**

In summary, this method has high sensitivity, specificity and on-site practicability for detecting GCRV type 1, and has great application potential in on-site GCRV monitoring.

## 1. Introduction

**Figure 1:**
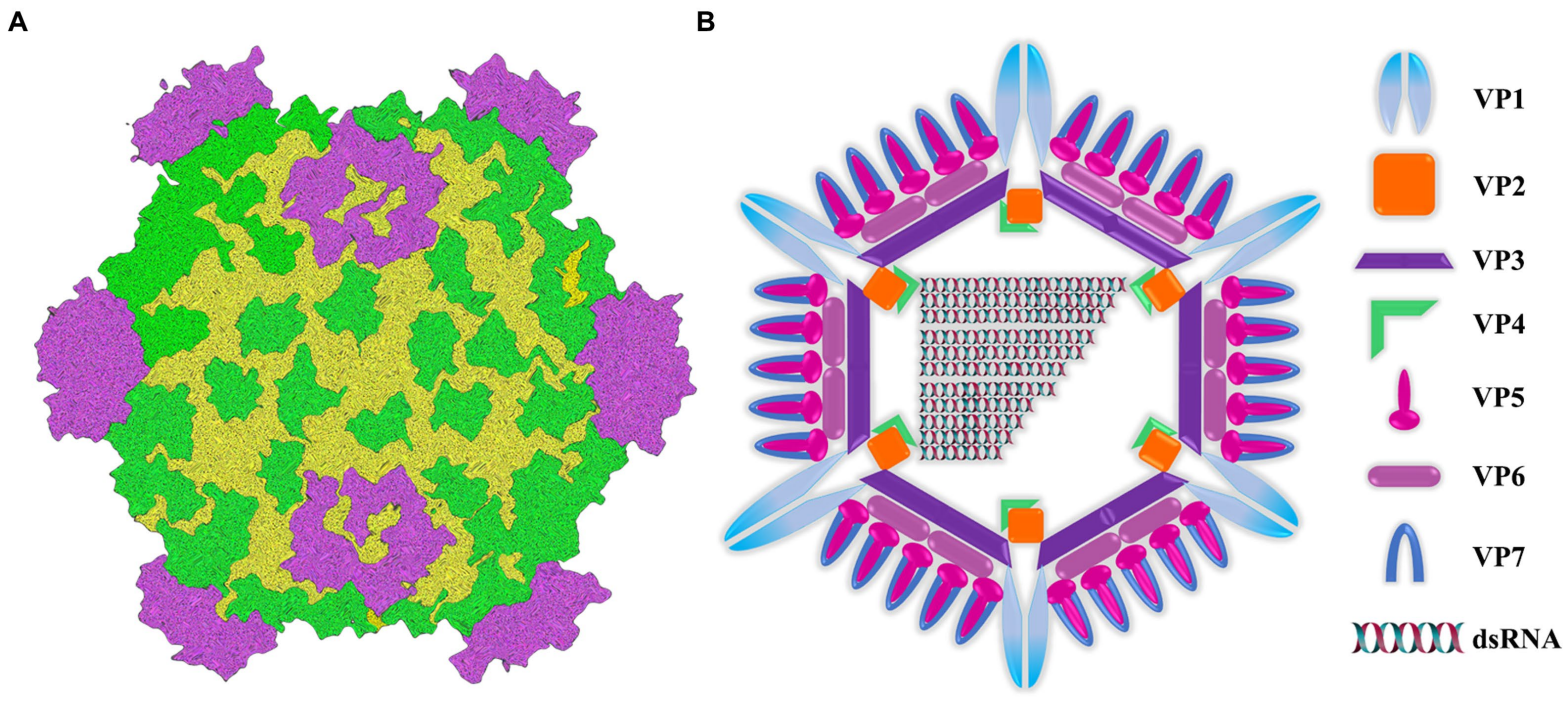
Image of intact virion and core particle of aquareovirus. **(A)** Three-dimensional reconstruction image of virus particles. **(B)** Viral structural gene diagram.

Aquatic reovirus belongs to a genus of the reovirus family, and is a vital pathogen in cultured aquatic animals. In particular, grass carp reovirus (GCRV) is considered to be the most pathogenic virus among the currently isolated aquatic viruses (Rangel et al., 1999; King et al., 2012; Zhang et al., 2019). From the perspective of genome structure, GCRV genome is composed of 11 segments of double-stranded RNA (Figure 1A). These segments are contained within a central core and are enveloped by a double-layered icosahedral capsid. The 11 genomic fragments encoded 7 structural proteins (VP1-VP7) and 5 non-structural proteins (NS80, NS38, NS31, NS26 and NS16) (Figure 1B) (Attoui et al., 2002; Zhang et al., 2010). The outer capsid of GCRV is composed of VP5-VP7 protein complex (Fang, 2005; Cheng et al., 2010; Yan L. et al., 2014), which is regarded to be involved in cell entry. In addition, inner capsid proteins VP1-VP4 and VP6 constitute the core of aquatic reovirus and are related to viral replication (Fang, 2005; Cheng et al., 2008). Due to the high stability of VP7 protein, *vp7* gene has become an extensively used target in nucleic acid detection and phylogenetic analysis (Fang et al., 2008; Zhang et al., 2010). After GCRV appears, it has transformed from being found exclusively in freshwater and saltwater to being isolated from a diverse range of aquatic reoviruses (Winton et al., 1987; Subramanian et al., 1994; Skoge et al., 2019; Fang, 2021). This has posed a significant threat to the reproduction of grass carp and even the aquaculture industry. Usually, GCRV-infected grass carp mainly shows severe intestinal bleeding, a very small amount of muscle bleeding and a certain number of intestinal muscle mixed bleeding (Wu et al., 2020). Currently, there is no optimal approach for treatment, and thus prevention is important. The main focus should be put on implementing effective prevention strategies. Therefore, it is necessary to establish an on-site diagnosis method to rapidly and accurately detect GCRV, which helps scientifically and effectively prevent and control virus spread and deterioration.

At present, numerous approaches are established to detect GCRV, among which, the earliest and most intuitive and effective methods include electron microscopic observation and cell culture (McEntire et al., 2003). Nevertheless, electron microscopy has high requirements for instruments and equipment, and the sample processing process is complicated. While cell culture for GCRV strains involves several rounds of infection and is time-consuming. Due to the unique characteristics of these isolates, complete cytopathic effect (CPE) on cells cannot be achieved, and visual observation is impossible. Therefore, virus isolation may not be an effective approach to prevent and control disease. In fact, GCRV is usually diagnosed on the basis of viral genome and antibody detection. If viral antibody is detected, the majority of serological detection methods are conducted on the basis of enzyme-linked immunosorbent assay (ELISA) due to its straightforwardness and cost-effectiveness (Zeng et al., 2018). However, its sensitivity is limited in subacute or early infections because of the extended time it takes for antibody production after viral infection. In addition, immunoblotting using specific antibodies is another method for virus detection (Jing et al., 2014), but it also suffers from the drawback of low sensitivity. Consequently, tools for detecting the GCRV genome, including polymerase chain reaction (PCR), real-time fluorescence quantitative PCR, or loop-mediated isothermal amplification (LAMP), have gained popularity because they are highly sensitive and specific in diagnosing GCRV (Zhang et al., 2010; Zeng et al., 2013; Zhang et al., 2013; Jing et al., 2014). Nonetheless, a main challenge with such technologies lies in their requirements of sophisticated equipment and trained professional manipulations, which hinders their practical application.

Microbial regularly clustered interspaced short palindromic repeats (CRISPR) and CRISPR-associated (Cas) systems are capable of recognizing and cleaving certain nucleic acid sequences (that is, by cis-cleavage). Cas13a, referred to C2c2 as well, is the RNA-targeting endonuclease (Makarova and Koonin, 2015). Additionally, CRISPR-Cas13a is used for detecting RNA targets and Cas13a collateral cleavage activity using the CRISPR-derived RNA (crRNA) (Gootenberg et al., 2017; Li et al., 2019; Wang et al., 2019). Based on recombinase polymerase amplification (RPA), T7 transcription of amplified DNA to RNA, together with CRISPR-Cas13a associated side effects, the Cas13a-based molecular detection platform SHERLOCK is constructed (Hao et al., 2017). This CRISPR-Cas13-based platform is successfully used for detecting various molecules such as Ebola virus (EBoV), severe acute respiratory syndrome coronavirus 2 (SARS-CoV-2), Zika virus (ZIKV) and Dengue virus (DENV) (Barnes et al., 2020; Crone et al., 2020; Li et al., 2020; Wang et al., 2021; Gaoxing et al., 2022).

Given the convenient and fast characteristics of the Cas13a-based molecular detection platform, its mature application in other virus detection fields, and the current gap in detecting GCRV, a detection system was established through the combination of CRISPR/Cas13a, RPA with lateral flow dipstick (LFD) to develop a simple way to detect GCRV in the present work. To achieve this, specific primers and probe combinations targeting the *vp7* gene conserved sequence were prepared, the system sensitivity was optimized, and the specificity was verified by clinical samples. Furthermore, the features of this system were utilized to suggest three methods of result interpretation, namely, collection of fluorescence signals, ultraviolet excitation visual fluorescence, and test strips. We are confident that this approach has the potential to be an alternative on-site detection method for GCRV, which enables timely monitoring and the swift development of the prevention and control strategies.

## 2. Materials and methods

### 2.1. Viral nucleic acid samples

The GCRV sample types and numbers detected in the present work are listed as follows. Field samples included 6 parts of epidermis, 7 parts of gill, 9 parts of brain, 8 parts of intestine and 7 parts of kidney, which were obtained from the Laboratory of Animal Important Pathogen Biology, College of Animal Science, Yangtze University. The viral genomic DNA/RNA extraction kit (Tiangen, Beijing, China) was utilized to extract viral genomic RNA and the one-step reverse transcription kit (Tiangen, Beijing, China) was adopted for cDNA synthesis.

### 2.2. RPA primer design and crRNA preparation

The *vp7* gene sequence of type I GCRV was chosen to be reference in designing primers. In addition, the nucleotide sequence of the *vp7* gene of GCRV was compared to determine the conserved region. In line with RPA primer design requirements, RPA primers were chosen from conserved nucleotide region in *vp7* gene. Moreover, T7 promoter sequence (GAAATTAATACGACTCACTATAGG) was ligated to 5′-end of RPA upstream primer. There were five primer sets being prepared by Sangon Biotech (Shanghai, China) (Table 1).

**Table 1:** Primer sequences used for *vp7* gene amplification.

Name		Sequence (5′-3′)
Primer 1	*vp7*-F1	GAAATTAATACGACTCACTATAGGGTGCCACTTCACATGATTCCGCAAGTCGCCCACGC
	*vp7*-R1	AGGCGCAGATGGTGTACCGCCCGCAGGTGACG
Primer 2	*vp7*-F2	GAAATTAATACGACTCACTATAGGGCACACGCTACCGCCGTAGTTGCCACCGCCGCT
	*vp7*-R2	TCCACATGCAAGTCGAGTCCAACGATCTGGCCGCA
Primer 3	*vp7*-F3	GAAATTAATACGACTCACTATAGGGGACAAGGTGCTGACTCGCTGCCCCAAGACT
	*vp7*-R3	TCATCCACGTTCGCCGATGTAAGGAGATCA
Primer 4	*vp7*-F4	GAAATTAATACGACTCACTATAGGGCGTCAGCCAAATGAAGCCATTCGCTCCTTAGTCG
	*vp7*-R4	GAGTCAGCACCTTGTCGTGGGGATGCGTATCGTC
Primer 5	*vp7*-F5	GAAATTAATACGACTCACTATAGGGCGCACTGTGGACTATCACGAATTGGATGTCAAAGCC
	*vp7*-R5	CAGGTGGAGTGGCAAGCAAAGGTCAGGTTGCTGAAC

**Figure 2:**
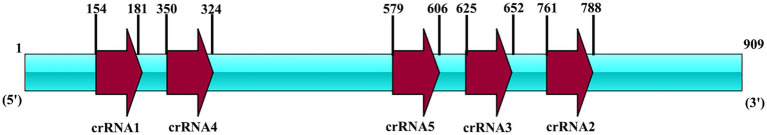
The locus diagram of each crRNA on the *vp7* gene. The *vp7* gene is 909 bp in length. CrRNA1 starts at 154 and ends at 181. CrRNA2 starts at 761 and ends at 788. CrRNA3 starts at 625 and ends at 652. CrRNA4 starts at 350 and ends at 324. CrRNA5 starts at 579 and ends at 606.

Moreover, five Cas13a crRNAs were designed for RPA product of *vp7*. In the preparation of crRNA, T7 promoter sequence was first cloned into the DNA template in crRNA, with all primers being prepared in Sangon Biotech (Table 2) (Figure 2). By adopting annealing buffer, two pairs of primers were annealed into double-stranded DNA, while the latter was then purified through gel recovery. In line with HiScribe T7 rapid high-yield RNA synthesis kit protocols, overnight incubation of double-stranded DNA under 37°C and transcription into crRNA were completed. According to manufacturer’s instructions, crRNA was purified with a spin-column RNA removal and concentration kit from Sangon Biotech (Shanghai, China). At last, crRNA was preserved under −80°C.

**Table 2:** Primer sequences used for crRNAs amplification.

Name	Sequence (5′-3′)
crRNA 1	GAUUUAGACUACCCCAAAAACGAAGGGGACUAAAACGUUAGUAGUCUCAGUUUUGGUUUUUGUG
crRNA 2	GAUUUAGACUACCCCAAAAACGAAGGGGACUAAAACCAGGUGGAGUGGCAAGCAAAGGUCAGGU
crRNA 3	GAUUUAGACUACCCCAAAAACGAAGGGGACUAAAACCUACGGCGGUAGCGUGUGCGUGAGUGUC
crRNA 4	GAUUUAGACUACCCCAAAAACGAAGGGGACUAAAACUUGACAUCCAAUUCGUGAUAGUCCACAG
crRNA 5	GAUUUAGACUACCCCAAAAACGAAGGGGACUAAAACAGUCUUGGGGCAGCGAGUCAGCACCUUG

The underlined area is the primer target sequences.

### 2.3. Standard plasmid preparation

The GCRV *vp7* gene fragment (GenBank: AF236688) was synthesized and subcloned into pET-28a (+) vector to construct the pET-28a-*vp7* plasmid utilized to be the detection target. Thereafter, the recombinant plasmid was transfected in *E. coli DH5α* prior to identification through enzyme digestion as well as DNA sequencing.

### 2.4. Evaluation and optimization of the RPA reaction

An RPA kit was purchased from Anhui Microanaly Genetech Co., Ltd. (Hefei, China). In this study, RPA reaction was carried out in line with RPA kit instructions. Specifically, the reaction system (50 μL) included buffer A (25 μL), nuclease-free water (13.5 μL), DNA sample (4 μL), magnesium acetate (2.5 μL) *vp7*-RPA-F (2 μL, upstream primer, 10 μM), *vp7*-RPA-R (2 μL, downstream primer, 10 μM) and RPA polymerase (1 μL). Different primer pairs were examined for screening the optimal primer pairs. Thereafter, RPA reaction product was obtained for CRISPR/Cas13a cleavage experiment.

### 2.5. CRISPR/Cas13a detection reaction

After preliminary optimization of the reaction and referring to the recommended system in the kit and published literature (Wei et al., 2022), CRISPR/Cas13a was detected using LwaCas13a (45 nM), crRNA (22.5 nM), RNA report (125 nM), dNTP (1 mM), RNase inhibitor (1 μL), RPA amplification product (1 μL), and T7 RNA polymerase (0.6 μL). LwCas13a was purchased from Anhui Microanaly Genetech Co., Ltd. (Hefei, China). The RPA reaction product samples were replaced by water without nuclease as the negative control. After incubation for a 40-min period under 37°C, the first method was to use a microplate reader to determine the relative FAM fluorescence signal intensity in the detection product. The second way was to place the product under the ultraviolet imaging system, so that the whole system emitted green fluorescence under the ultraviolet light. The third method was to utilize the lateral flow test strip for determining, transferring CRISPR/Cas13a detection product (10 μL) in detection buffer (90 μL), and inserting the strip. Following 5-10-min incubation, a digital camera was utilized for observing and recording specific bands.

### 2.6. RPA-CRISPR assay

**Figure 3:**
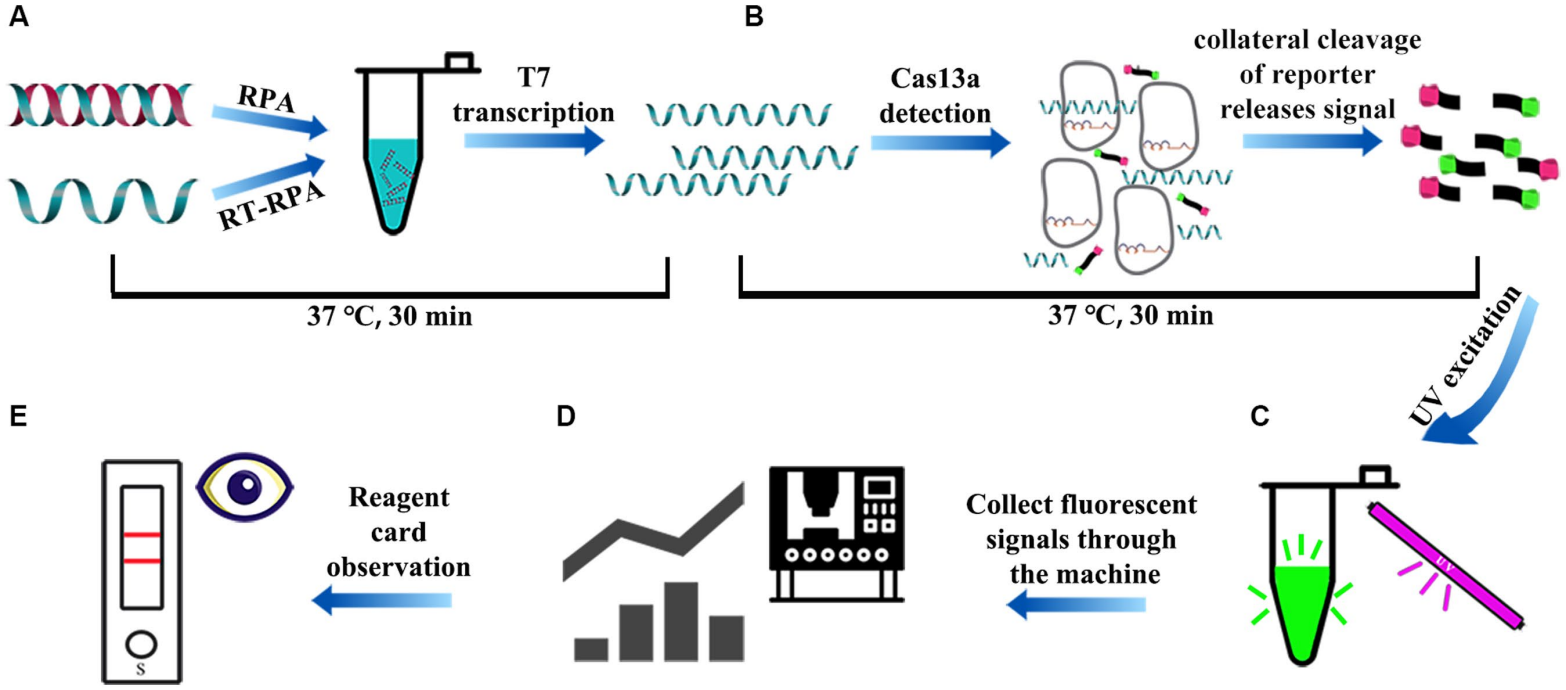
The schematic diagram of the whole process of RPA-CRISPR/Cas13a detection. **(A)** After rapid treatment of the sample within 10 min, the extracted DNA/RNA was subjected to RPA/RT-RPA amplification of the target sequence (15–30 min). **(B)** The *vp7* gene in the RPA product was amplified by RPA and transcribed into RNA, which was recognized by crRNA to activate Cas13a nuclease. Activated Cas13a nuclease then cleave the reporter molecule (15 ~ 30 min). **(C)** Visible green fluorescence detection of RPA-CRISPR/Cas13a system under UV light. **(D)** The fluorescence signal intensity of RPA-CRISPR/Cas13a system was detected by microplate reader. **(E)** Reporter molecules can appear in the form of bands on the test strip.

The RPA-CRISPR/Cas13a detection procedure included RPA amplification of dsDNA template and the optimized Cas13a cleavage experiment. In brief, genome extraction kit was used to extract the genome of tissue and other samples for reverse transcription, then RPA reaction system was introduced onto the PCR tube bottom, and the freeze-dried powder of Cas13a active protein was placed in the tube cap. The reaction was first subjected to 30-min incubation under an optimal RPA reaction temperature, the target genes were amplified by RPA and transcribed into RNA by T7 transcriptase. RNA was recognized by crRNA to activate Cas13a nuclease, and activated Cas13a nuclease then cleaved the reporter molecule (30 min) (Figure 3A). After thorough centrifugation of PCR tube, RPA and Cas13a reactions were blended (Figure 3B). The blended reaction was performed, and fluorescence was observed with an ultraviolet imaging system (Figure 3C). In addition, the PCR tube system fluorescence intensity was detected by the microplate reader (Figure 3D). The strip was visually observed on the test strip (Figure 3E).

The pET-28a-*vp7* plasmid was diluted from 7.2 × 10^5^ copies/μL series to 7.2 × 10^−1^ copies/μL, and the limit of detection was determined. With regard to RPA-CRISPR/Cas13a method, its sensitivity was tested by using diverse plasmid dilutions as templates and amplification products by RPA reaction. At the same time, the *vp7* fluorescence quantitative primers q-*vp7*-F: CAAGACCATTCAAGACTC and q-*vp7*-R: TCACTCACTTCGACTAAT were synthesized for comparison.

For further determining the specificity of the established RPA-CRISPR/Cas13a detection method, more than 10 common aquatic pathogens were detected. These pathogens included infectious spleen and kidney necrosis virus (ISKNV), Channel catfish virus (CCV), Largemouth Bass Virus (LMBV), Cycarp herpesvirus (CYHV), Viral Hemorrhagic Septicemia Virus (VHSV), *Aeromonas hydrophila* (Ah), *Aeromonas sobria* (As), *Aeromonas veronii* (Av), *Edwardsiella tarda* (Et), *Klebsiella Pneumoniae* (Kp), *Plesiomonas shigelloides* (Ps). The above pathogens were detected and preserved in the laboratory of animal important pathogen biology, College of Animal Science, Yangtze University.

### 2.7. Comparison of RPA–CRISPR/Cas13a with real-time qPCR from clinical samples

For further verifying the method reliability, the coincidence rate of CRISPR/Cas13a versus qPCR was analyzed in 39 clinical samples. To be specific, the qPCR detection of *vp7* gene was performed using the Polyme fluorescence quantitative kit, and the primers are shown in 2.7. The reaction system (25 μL) included 2x M5 HiPer SYBR Premix EsTaq (Beijing Mei5bio, 12.5 μL), DNA (2 μL), primer F (0.5 μL), primer R (0.5 μL), and ddH_2_O. Meanwhile, reaction cycle parameters were set to 30-s denaturation under 95°C; 5-s amplification under 95°C and 30-s under 60°C for 40 cycles. In the meanwhile, qPCR was carried out in parallel using cDNA isolated in these samples.

### 2.8. Statistical analysis

GraphPad Prism 8.0 (GraphPad Software, Inc.) was employed for data analysis with analysis of variance (ANOVA). Values were represented by means ± SD from three separate assays. *p* < 0.05 stood for statistical significance.

## 3. Results

### 3.1. Detection of RPA-amplified products and establishment of the CRISPR/Cas13a

**Figure 4:**
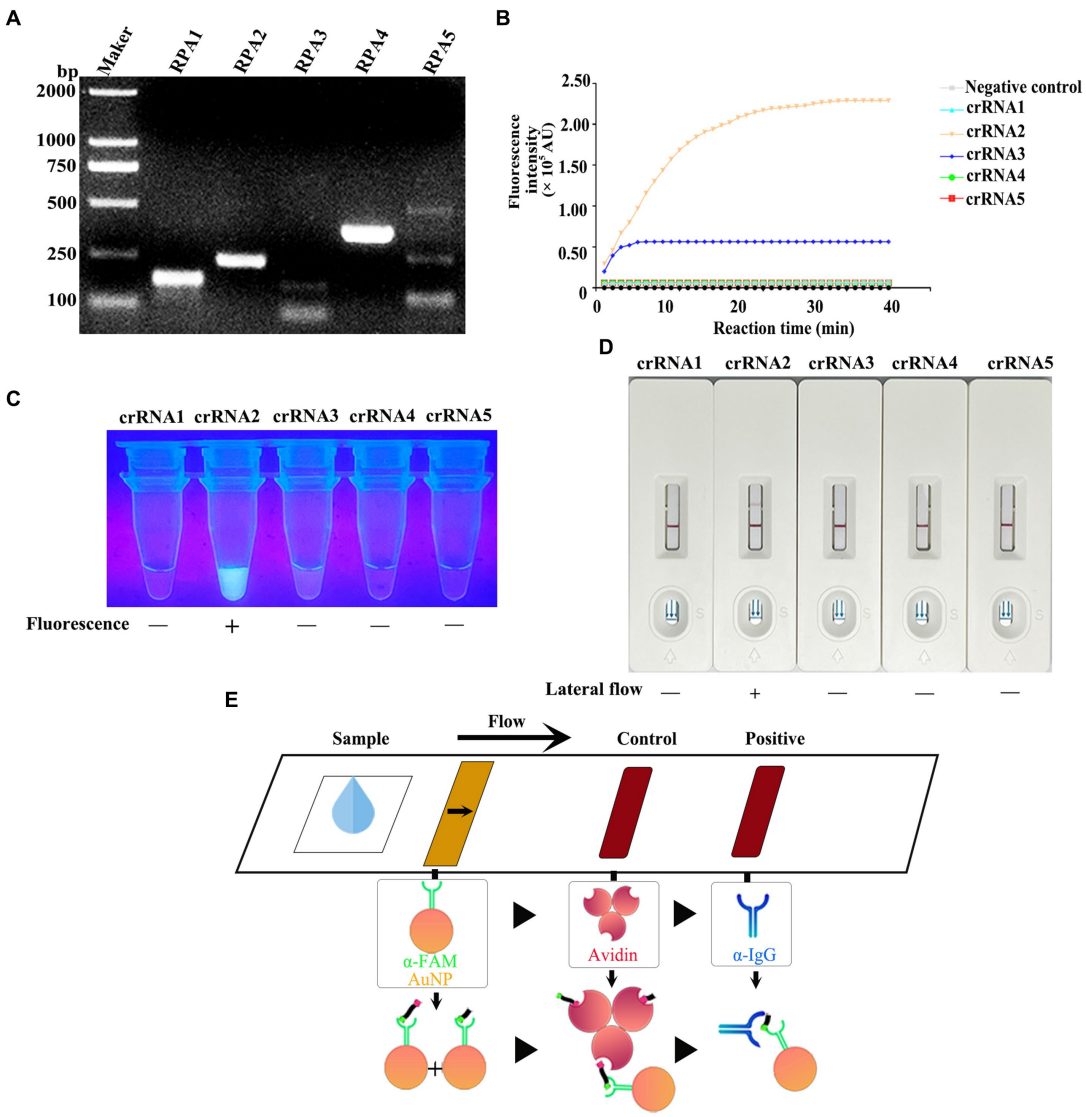
**(A)** After the RPA reaction was carried out according to the instructions of the RPA kit, the product was detected by 1% agarose gel electrophoresis (80 V, 30 min). **(B)** CRISPR/Cas13a system was prepared using RPA amplification products as templates, and the fluorescence intensity of the reaction system was detected in real time at 37°C using a microplate reader. **(C)** The products that completed the CRISPR/Cas13a cleavage experiment were placed under ultraviolet light. **(D)** The products of the CRISPR/Cas13a cutting experiment were determined by the test strip. **(E)** Schematic diagram of test strip principle. For the negative samples, the gold-labeled NP-anti-FAM antibody was fully coupled with the RNA reporter molecule, and the conjugate was blocked by streptavidin in the control band, so that only one band could be observed on the C line. For positive samples, the results depend on the efficiency of crRNA cleavage. If the cutting efficiency is high, the RNA reporter gene is completely cut, and a high-intensity band is observed at the T line of the band, and no band is observed at the C line. In contrast, if the RNA reporter gene is not completely cut, bands can be observed at the T-line and C-line of the band, and the strength of the band depends on the cutting efficiency.

For ensuring specificity and efficient amplification, five RPA primer sets were synthesized according to the *vp7* gene conserved nucleotide region. Besides, agarose gel electrophoresis (AGE) and NanoPhotometer N60 microspectrophotometer (Simplen, Germany) were utilized to detect fragment size and content of RPA products, and qualified primers were screened. All the corresponding RPA products had electrophoresis bands close to the theoretical size, among which, Primer 1, Primer 2 and Primer 4 bands were clear and displayed good specificity. Primer 3 and Primer 5 had the theoretical size bands, but there were miscellaneous bands (indicating non-specific amplification) (Figure 4A), and product content met the later detection experimental requirements (Table 3). Consequently, these primers were chosen and evaluated in later experiments.

**Table 3:** Amplified products use NanoPhotometer N60 detection results.

Primer Number	Product Concentration (ng/μL)	Theoretical Product Size (bp)
RPA-Primer 1	51	150
RPA-Primer 2	54	214
RPA-Primer 3	35	140
RPA-Primer 4	52	346
RPA-Primer 5	46	470

Five corresponding crRNAs were designed and prepared for five pairs of RPA primers. To screen the effective RPA/crRNA combinations, RPA product was used as a template, and a microplate reader was used to perform a reaction at 37°C. The fluorescence signal value was collected in a real-time manner, and the curve was drawn over a period of time. As shown in Figure 4B, crRNA 2 and crRNA 3 displayed changes in the fluorescence signal value during the entire reaction process, with increases to varying degrees, while crRNA 2 had a higher signal value, reaching a peak at 30 min of the reaction. The remaining three groups of crRNA did not detect changes in signal values. Subsequently, the whole reaction system was placed under the ultraviolet imaging system, and it was found that crRNA 2 showed bright green fluorescence under the ultraviolet light excitation, crRNA 3 also had weak fluorescence emission, while the other three groups did not show visible fluorescence (Figure 4C). As a result, crRNA 2 was kept as a potential candidate.

To achieve on-site detection, RPA was combined with LwaCas13a and lateral flow dipstick to establish the CRISPR/Cas13a-LFD detection method. The FAM-RNA-biotin reporter gene was adopted for lateral flow dipstick detection. In addition, with regard to negative samples, the gold-NP-anti-FAM antibody was fully coupled to the FAM-RNA-biotin reporter gene, while the biotin ligand was used to intercept the conjugate in control band. In terms of positive samples, the cleavage of FAM-RNA-biotin reporter gene was completed, followed by accumulation of gold-NP-anti-FAM antibody-FAM conjugate in the detection band and reduction in the control band. Figure 4E displays the sketch map for lateral flow detection. For the RPA product of GCRV *vp7*, all crRNAs were selected for CRISPR/Cas13a-LFD detection. As observed from the band display, when the other four groups of crRNAs were not successfully colored with the test strip, crRNA 2 showed a clear positive detection band (Figure 4D). The combination of Primer 2 + crRNA 2 was successfully screened, and the RPA-CRISPR/Cas13a detection system was established.

### 3.2. Establishment of RPA-CRISPR/Cas13a

**Figure 5:**
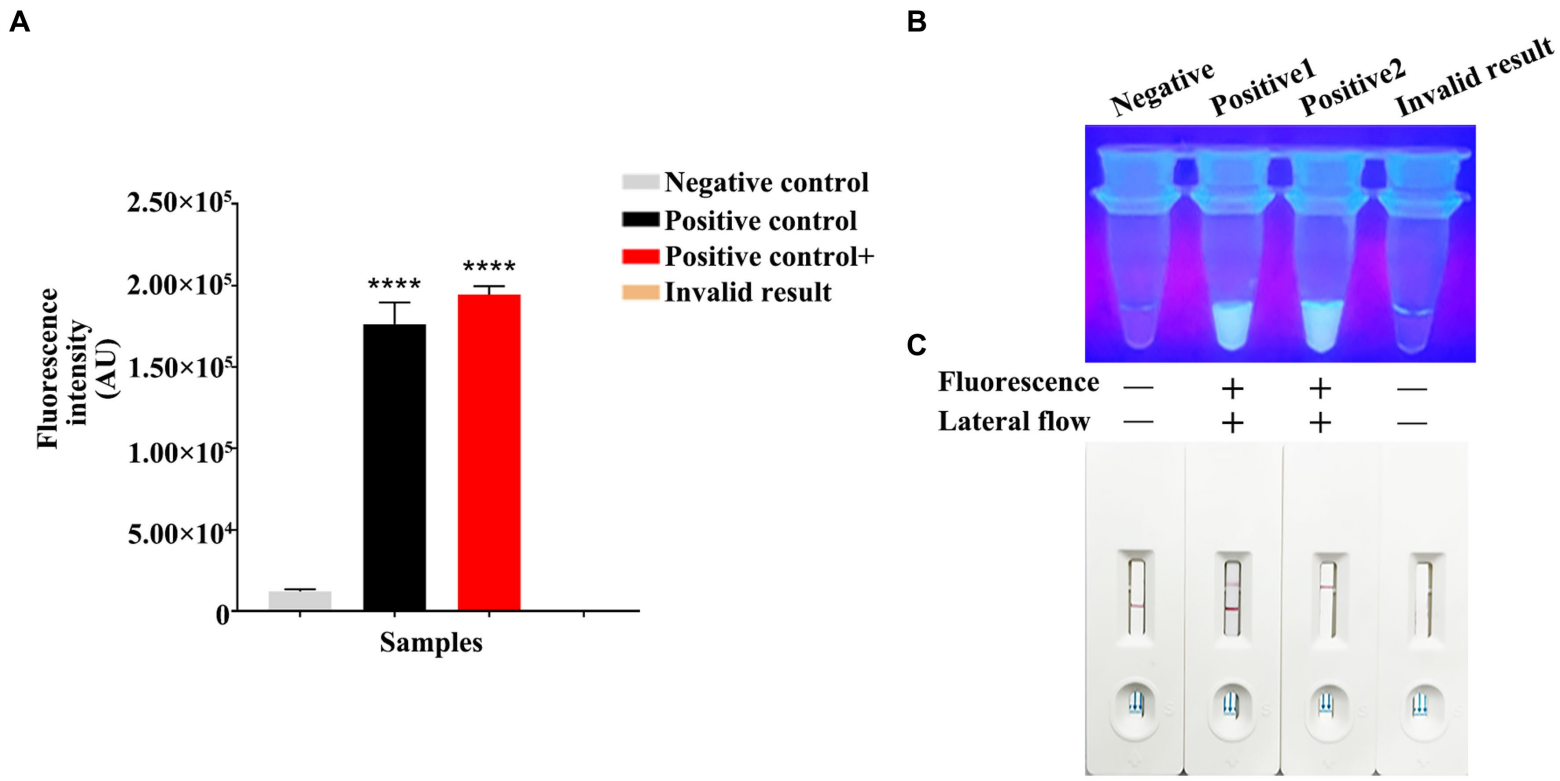
RPA-CRISPR/Cas13a results criteria. **(A)** Using a microplate reader, the negative fluorescence value under the FAM fluorescence channel is extremely low or undetectable. High fluorescence values can be detected in both positive/strong positive. If invalid, the fluorescence value cannot be detected. **(B)** Under ultraviolet light irradiation, negative/invalid did not emit fluorescence. Clear and bright green fluorescence can be observed in both positive/strong positive. Fluorescence clarity varies depending on vision and template copy number. **(C)** Negative results showed only red bands at the C line of the test strip. The positive results showed red bands on both C and T lines of the test strip. The strong positive results only appeared red bands in the T line, which appeared in the case of high reaction cutting efficiency and thorough reaction. The invalid results were not shown in red bands on the C and T lines.

Using the constructed pET-28a-*vp7* as a positive control, the genome was added into the RPA reaction on the PCR tube bottom, while the Cas13a reaction was carried out in the lid. The reaction procedure included the 30-min incubation under the optimal RPA reaction temperature. After thorough centrifugation of PCR tube, RPA and Cas13a reactions were blended. Then, product (4 μL) was introduced into the buffer solution (36 μL), followed by addition of the resultant mixture into the sample pad on the test strip. The test results were determined after standing for 5 min. The judgment criteria are shown in Figure 5C. Changes in T and C line colors of LFD test strip were observed. When both lines or just T line showed red color, the result was judged as positive. When just C line showed red color, it was judged as negative; while if T and C lines were colorless, the result was invalid. When the fluorescence intensity was measured by a microplate reader, both positive and strong positive results exhibited the strong fluorescence signals (Figure 5A). In the dark room, both positive and strong positive results emitted clear and bright green fluorescence under ultraviolet light (Figure 5B).

### 3.3. Sensitivity test of the RPA-CRISPR/Cas13a

**Figure 6:**
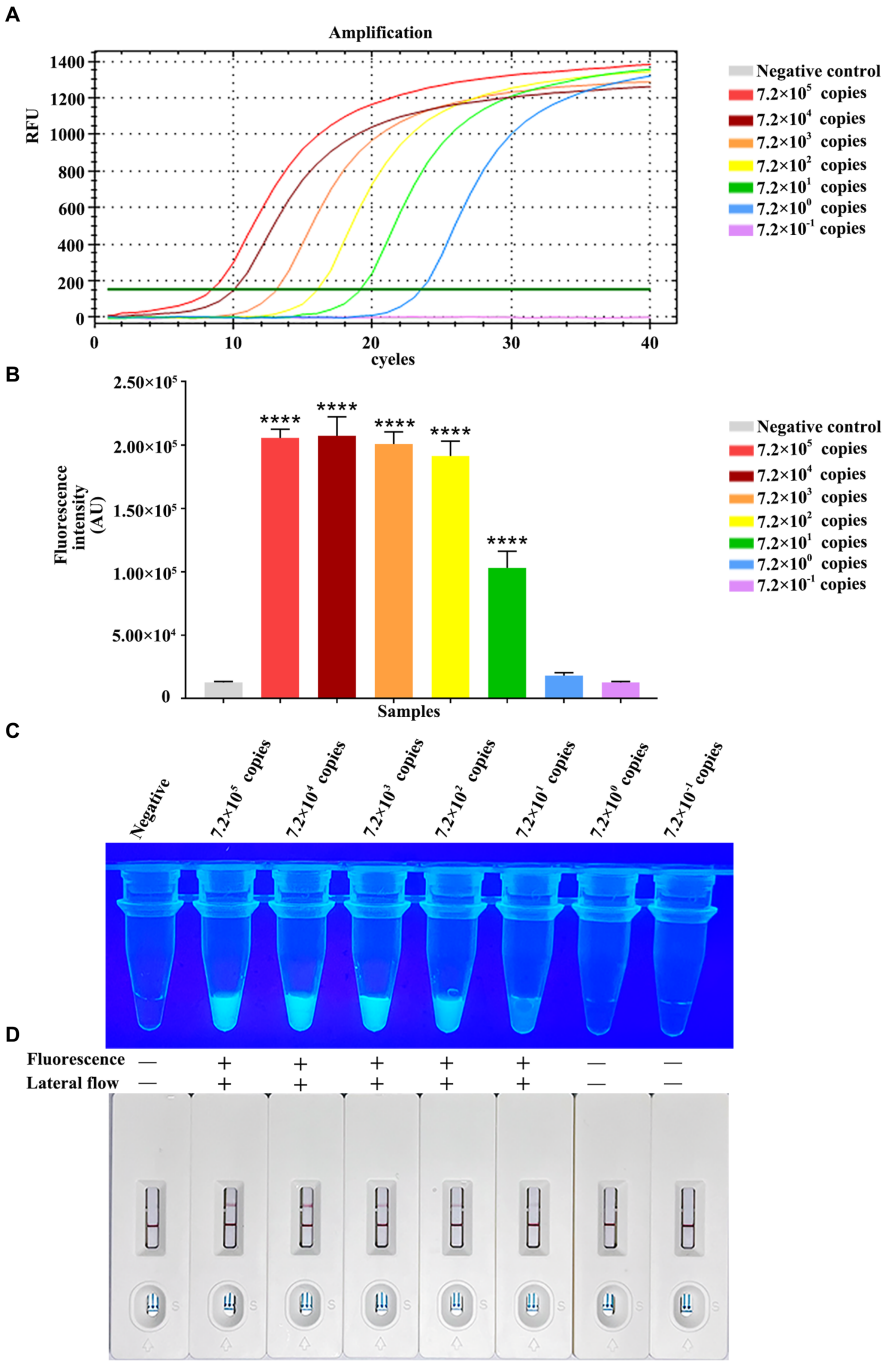
Analytical specificity of RPA-CRISPR/Cas13a. Sensitivity comparison between qPCR. **(A)** The qPCR amplification curve of *vp7* gene at different dilutions. **(B)** The fluorescence intensity of RPA-CRISPR/Cas13a at different dilutions of *vp7* gene was detected by microplate reader. **(C)** The results of RPA-CRISPR/Cas13a UV irradiation of *vp7* gene at different dilutions. **(D)** The results of RPA-CRISPR/Cas13a test strip of *vp7* gene at different dilutions.

For testing the method performance, we diluted constructed pET-28a-*vp7* by 10-fold serial dilution from 7.2 × 10^5^ copies/μL to 7.2 × 10^−1^ copies/μL for detecting the RPA-CRISPR/Cas13a method sensitivity based on the combination of Primer 2 + crRNA 2. At the same time, the fluorescence quantitative method of *vp7* gene was used as a control for comparison. The fluorescence quantitative results showed a standard amplification curve from 7.0 × 10^5^ copies/μL to 7.2 × 10^0^ copies/μL (Figure 6A), and the minimum limit of detection of the fluorescence quantitative method was 7.2 × 10^0^ copies of DNA/reaction.

Additionally, the RPA-CRISPR/Cas13a analysis sensitivity was analyzed through 10-fold serial dilution of plasmid, at concentrations of 7.2 × 10^5^–7.2 × 10^−1^ copies/μL/reaction. Fluorescence value was measured by a microplate reader, as a result, the reactions with 7.2 × 10^2^–7.0 × 10^5^ copies/μL plasmid generated strong fluorescence, with no potent relation of copy number with signal intensity. Moreover, 7.2 × 10^1^ copies/μL plasmid generated potent fluorescence as well, and there was difference in the intensity compared with negative control (7.2 × 10^0^ and 7.2 × 10^−1^ copies/μL) (Figure 6B). Under ultraviolet light, 7.2 × 10^1^ copies/μL plasmid still produced positive result, but no green fluorescence was observed in the tubes of negative control, and reactions with 7.2 × 10^0^ or 7.2 × 10^−1^ copies/μL plasmid (Figure 6C). When the same dilution series was analyzed with the RPA-CRISPR/Cas13a-LFD method, the limit of detection was determined to be 7.2 × 10^1^ copies/μL plasmid (Figure 6D). In conclusion, the above results indicated that the limit of detection of RPA-CRISPR/Cas13a-LFD method was 7.2 × 10^−1^ copies/μL plasmid/reaction, the same as that obtained under fluorescent and ultraviolet light.

### 3.4. Specificity test of RPA-CRISPR/Cas13a

**Figure 7:**
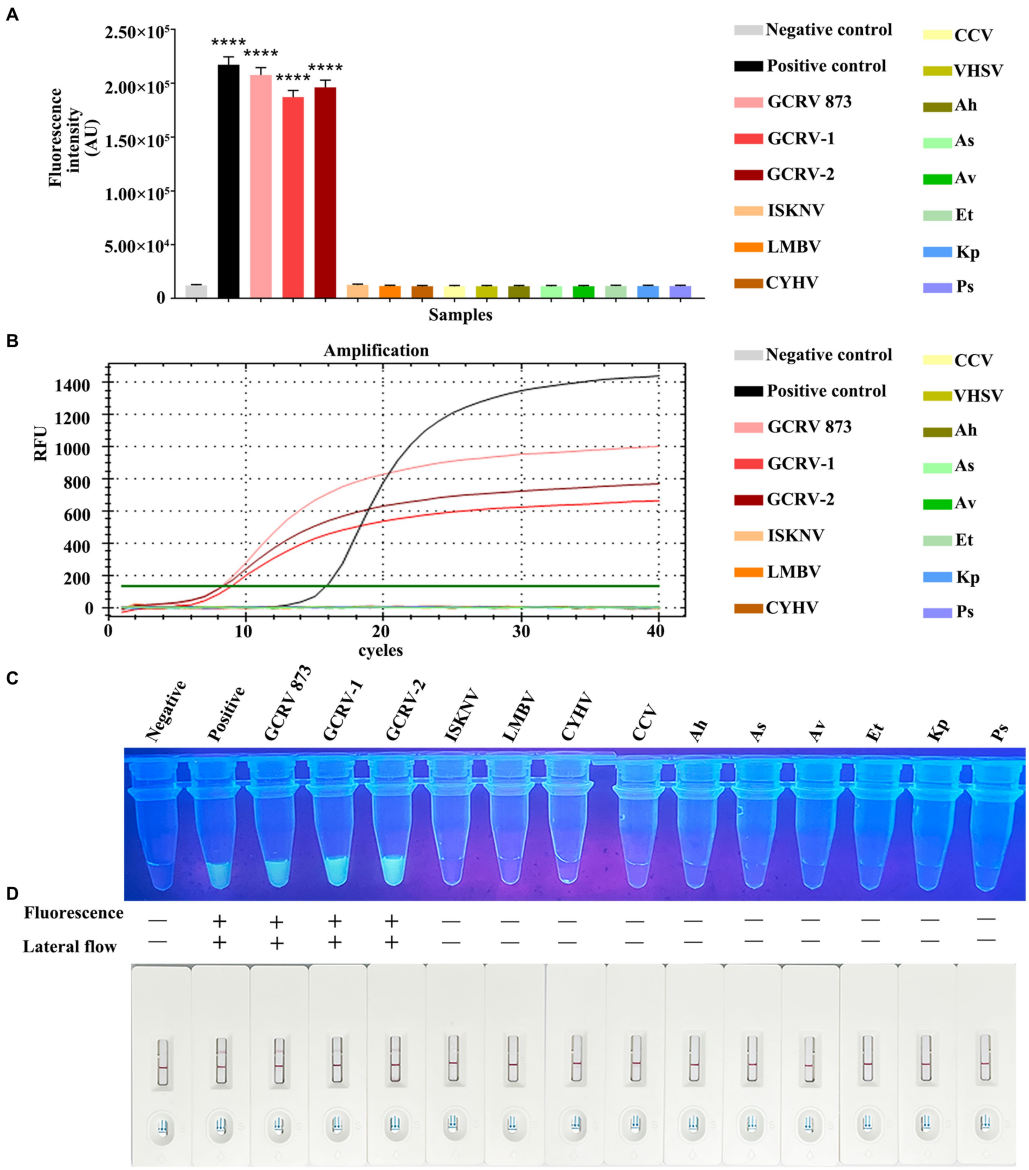
Specificity comparison between RPA-CRISPR/Cas13a and qPCR. **(A)** The fluorescence intensity of common aquatic pathogens detected by RPA-CRISPR/Cas13a was detected by a microplate reader. **(B)** The amplification curve of qPCR for common aquatic pathogens. **(C)** The results of RPA-CRISPR/Cas13a UV irradiation of common aquatic pathogens. **(D)** The RPA-CRISPR/Cas13a test strip results of common aquatic pathogens.

For evaluating whether RPA-CRISPR/Cas13a was specific, this method was adopted to detect additional common aquatic pathogens, which included ISKNV, LMBV, CCV, CYHV, VHSV, Ah, Av, As, Et, Kp, and Ps. The Primer 2 + crRNA 2 combination was selected for detection. Meanwhile, the fluorescence quantitative method of *vp7* gene was used as a control for comparison. Fluorescence quantitative results indicated that only GCRV showed a positive curve, while the remaining 11 pathogens did not exhibit a positive curve (Figure 7A). According to the fluorescence value measured by a microplate reader, there was no fluctuation of fluorescence signal value in the 11 other pathogens, while the positive fluorescence signal value was only detected in GCRV (Figure 7B). Under ultraviolet light, only the GCRV group emitted the clear and bright green fluorescence, while the other 11 pathogens did not emit green fluorescence (Figure 7C). Based on test strip results, all those 11 common aquatic pathogens showed negative bands on the test strips, while the positive bands were only observed on the test strips of GCRV (Figure 7D). Combined with fluorescence quantitative results, this method was highly specific in detecting GCRV.

### 3.5. Clinical samples detection by the RPA-CRISPR/Cas13a

For verifying that RPA-CRISPR/Cas13a was reliable in detecting GCRV clinical samples, RPA-CRISPR/Cas13a was used to analyze 39 nucleic acid samples collected from tissues through qPCR. According to Table 4, results of RPA-CRISPR/Cas13a in clinical samples were consistent with those of qPCR, suggesting a 92.31% coincidence rate between both methods. Using this method, 24 samples were detected to be positive for GCRV and 15 were negative. These positive results conformed to qPCR results. Consequently, the above findings indicate that RPA-CRISPR/Cas13a can accurately detect clinical GCRV on site with no need of costly equipment. In addition, the concentration of *vp7* gene in the positive samples ranged from 6.51 × 10^0^ copies/μL to 4.51 × 10^5^ copies/μL. When the CT value was 23.45 (the concentration of virus *vp7* gene was 6.80 × 10^1^ copies/μL), both fluorescence quantitative PCR and RPA-CRISPR/Cas13a method could detect the virus. However, when the concentration of *vp7* gene was lower than 6.80 × 10^1^ copies/μL, the sensitivity of RPA-CRISPR/Cas13a method was not as good as that of fluorescence quantitative PCR.

**Table 4:** The coincidence rate between the CRISPR/Cas13a-LFD and real-time qPCR for the GCRV detection in clinical samples.

Sample types	Number	Results (Positive/Negative)		
		RPA-LFD	qPCR	Concentration of vp7 gene(copies/μL)
Epidermis	6	2/6	3/6(CT = 21.18–34.15)	6.51 × 10^0^–1.05 × 10^5^
Gill	7	4/7	5/7(CT = 20.54–35.42)	5.96 × 10^1^–1.36 × 10^5^
Brain	9	6/9	6/9(CT = 17.43–36.35)	1.07 × 10^2^–2.80 × 10^5^
Kidney	9	7/9	7/9(CT = 14.24–34.77)	8.63 × 10^3^–4.29 × 10^5^
Intestinum	8	5/8	5/8(CT = 13.78–33.15)	2.23 × 10^1^–4.51 × 10^5^
In total	39	24/39	26/39	

## 4. Discussion

In China, grass carp represents a significant freshwater fish species, and its yield accounts for 20.69% of overall freshwater aquaculture yield (Li et al., 2022). Grass carp also has a wide range of cultures in more than 40 countries (Shen et al., 2019). However, the mortality rate is as high as 90% since Grass carp Hemorrhagic Disease (GCHD) frequently occurs, which greatly hinders the breeding of grass carp. Vaccination and early diagnosis is the efficient way for preventing and controlling GCHD (Brudeseth et al., 2013). Numerous techniques have been documented for identifying GCRV infection, such as electron microscopy, virus isolation, nucleic acid sequence amplification plus ELISA (NASA-ELISA), LAMP, and RT-PCR (Liu et al., 2012; Zeng et al., 2013; Wang et al., 2020). Nonetheless, the above technologies are time-consuming, labor-intensive, nonsensitive, impossible for quantification and need AGE of amplified product. Consequently, they are not suitable for rapid diagnostic procedures and may not be appropriate for epidemiological analyses. There is limited research assessing PCR-based GCRV quantification (Youliang and Jianguo, 2015). In the present work, a GCRV detection system with rapidness, sensitivity and specificity was constructed on the basis of CRISPR/Cas13a-based RPA and test strip. The proposed method can be conducted by individuals without specialized training. What the users need to do is adding a specific reagent into the tested nucleic acid sample, completing this reaction within the constant-temperature water bath, and later using a test strip to obtain visible results under the naked eye. Besides, the essential equipment for the test includes water bath pot and pipette gun. The detection reagent remains stable for over 6 months at temperatures between −15°C ~ −25°C, which shows its potential application value in GCRV field detection.

SHERLOCK (Specific High Sensitivity Enzymatic Reporter UnLOCKing) is a highly sensitive and specific nucleic acid detection technology developed by combining RPA with clustered regularly interspaced short palindromic repeats (CRISPR)/CRISPR associated protein 13a (CRISPR/Cas13a). According to whether RT-RPA nucleic acid amplification and CRISPR/Cas13a detection are combined into the same reaction system, it is divided into two-step SHERLOCK and one-step SHERLOCK. The two-step SHERLOCK method is more sensitive, and the one-step SHERLOCK method is less time-consuming, less risk of sample contamination, and easier to obtain quantitative test results (KroLov et al., 2014; Zhao et al., 2015; Prakash et al., 2016). SHERLOCK is widely used in the detection of various pathogens due to its unique technical characteristics. Jon Arizti-Sanz et al. combined one-step SHERLOCK with rapid pyrolysis of samples at room temperature, reagent freeze-drying process, and side-stream chromatography test strips to provide a simple and rapid new coronary diagnosis for areas far from medical centers. The protocol, from nucleic acid extraction to detection completion, takes only 90 min, and the sensitivity reaches 200 copies/μL (Xue et al., 2020). In addition, SHERLOCK was also applied to the detection of Zika virus, Ebola virus and influenza A virus H7N9 (KroLov et al., 2014; Lobato et al., 2018; Salviato et al., 2018). Regardless of the fact that the nucleic acid extraction process has the same reaction time as the existing SHERLOCK, the detection method on the basis of CRISPR/Cas13a-based RPA and test strip established in this study has better sensitivity than the SHERLOCK established by Jon Arizti-Sanz et al.

In 2018, Chen et al. developed an accurate and rapid assay using Cas12a. This method combines Cas12a with isothermal amplification, named DETECTR (DNA endonuclease-targeted CRISPR trans reporter), and achieves highly sensitive detection of human papillomavirus (HPV) in patient samples (Chen et al., 2018). Cas12a can self-process pre-crRNA into mature crRNA without the assistance of RNA or other proteins, so Cas12a can achieve the cleavage of target sites without tracrRNA. In addition, Cas12a has ssDNA non-specific cleavage activity, also known as trans-cleavage activity. The platform combines CRISPR/Cas system with RPA constant temperature amplification technology, which can efficiently detect a variety of pathogenic microorganisms. Cas12a has high sensitivity and practical value in detecting DNA. It is simple, fast and does not require large-scale instruments and equipment. However, compared with other CRISPR detection systems, it cannot be used for single-base difference detection, and the accuracy needs to be improved. Cas13a, also known as C2C2, recognizes and cleaves target ssRNA under the guidance of crRNA. Similar to Cas12a, it has the ability to cleave any sequence of ssRNA in a non-specific system (Gootenberg et al., 2017). Combining Cas13a with RPA isothermal amplification technology, a Cas13a-mediated RNA detection platform: SHERLOCK was developed. This detection method detected Zika virus and dengue virus, and also achieved ideal results in tumor disease detection (Salviato et al., 2018). In the detection of double-stranded DNA, Cas12a can be preferred, and Cas13a can be preferred in the detection of RNA. The detection method established by combining CRISPR/Cas system with RPA technology has the advantages of high resolution and high sensitivity, low cost and simple operation. It has great application prospects in the fields of disease diagnosis, environmental assessment, food safety and so on.

As the RPA-CRISPR/Cas13a specificity is determined by mismatch number within the crRNA target nucleotide region, GCRV *vp7* sequences of diverse genotypes were compared (Hao et al., 2017). According to alignment analysis, we synthesized five RPA primer sets together with relevant crRNAs into the conserved nucleotide region of *vp7* gene of GCRV. Based on the preliminary detection using 5 primer-probe combination pairs, the primer-probe combination with highest sensitivity was selected for optimization.

A microplate reader and ultraviolet imaging system are applied in accurately judging test results in the laboratory. Typically, RNA-targeting CRISPR effector CRISPR-associated 13 showed a collateral effect of mismatched ribonuclease activity in recognizing targets. After the binding of LwaCas13a crRNA to target RNA, the LwaCas13a cis-cleavage activity can be activated and non-target RNA is degraded. In the present work, LwaCas13a para-cleavage activity was used. When Cas13a was cut in the reaction system, the quenching group in the reaction system was cut, and its connected fluorescent group FAM emitted green fluorescence. The relative fluorescence intensity value was measured under the microplate reader, and the positive product under the ultraviolet light showed green fluorescence.

Lateral flow test strips were adopted for on-site test result analysis (Abudayyeh et al., 2017). For the sake of preventing false positive results while controlling production expenses, the optimal streptavidin content within test strip was determined based on RNA reporter volume utilized for an individual reaction. With regard to the negative samples, we fully coupled the gold-labeled NP-anti-FAM antibody with RNA reporter molecule, then blocked the conjugate using streptavidin in control band, so that just one band was seen on C line. Results of positive samples were determined by crRNA cleavage efficiency (Wang et al., 2019). RNA reporter gene can be totally cleaved in the case of high cutting efficiency, and a high-intensity band can be seen on T line, but no band can be seen from C line. In contrast, if RNA reporter gene cannot be totally cut, bands are seen on T and C lines, with band intensity being determined by cutting efficiency.

In this study, the limit of detection of RPA-CRISPR/Cas13a reached 7.2 × 10^1^ copies/μL, indicating that RPA-CRISPR/Cas13a was sensitive to GCRV detection. RPA-CRISPR/Cas13a conventional qPCR achieved similar sensitivity and accuracy. Meanwhile, this method had no cross-reaction with more than 10 common pathogens of aquatic products, proving its good specificity and great significance in clinical detection. For verifying that RPA-CRISPR/Cas13a was reliable in detecting GCRV, the numbers of GCRV positive samples and GCRV negative samples were analyzed by qPCR, then the coincidence rate of RPA-CRISPR/Cas13a with qPCR was determined. According to these findings, RPA-CRISPR/Cas13a had the potential to be utilized for on-site GCRV detection, and its coincidence rate was 92.31% for GCRV positive and negative samples. The results obtained from RPA-CRISPR/Cas13a analysis of clinical samples were 92.31%, conforming to those obtained from fluorescence quantitative PCR. We speculated that this might be due to the lower viral genome copy number in clinical samples than the limit of detection of RPA-CRISPR/Cas13a. Our main objective now is to explore methods for enhancing assay sensitivity. This technique can serve as a basis for future research in this field.

Genomic sequence analysis and protein structural 3D reconstruction revealed that GCRV and additional reoviruses shared similar structures to water viruses, yet numerous differences should also be noted, probably caused by genus-specific differences associated with virus-host interactions. According to sequence analysis, the genomic fragment S10-encoded outermost VP7 capsid proteins were all structural proteins (Attoui et al., 2002; Cheng et al., 2008). The genome of GCRV consists of 11 segments of dsRNA. The seven structural proteins encoded by the virus are defined as VP1-VP7 according to the molecular weight. VP7 is one of the main capsid proteins, and the viral capsid contains 200 trimers composed of VP5/VP7 heterodimers (Liu et al., 2017). VP7 protein is a GCRV-specific outer capsid protein and plays an important role in GCRV infection and pathogenesis (Fang et al., 2008). VP7 can bind to dsRNA, which is related to the cell adsorption of the virus (Benavente and Martínez-Costas, 2007), especially plays a key role in the interaction between the virus and the host and the entry of the virus into the cell (Yan X. Y. et al., 2014). The trimer on the outer capsid exists as a receptor recognition site, so it is speculated that VP7 protein is involved in the process of GCRV invading grass carp cells (Shao et al., 2011). Chen et al. (2013) found that the antiserum produced by grass carp infected with GCRV can only recognize the outer capsid protein VP7 of GCRV-I, indicating that *vp7* is the main antigen protein of GCRV. The above results suggest that the *vp7* gene plays an important role in viral infection and replication.

Therefore, *Vp7* was selected as target gene to detect RPA-CRISPR/Cas13a.

GCRV can be divided into GCRV I, GCRV II and GCRV III. At present, the three genotypes of GCRV have been deeply explored. The three GCRV genotypes often show some differences in the genome after their own evolution. Therefore, there are many selection options when identifying GCRV genotypes in the published detection methods or standards, among which S6 segment is the most common (Weiwei et al., 2013). *Vp5* and *Vp7* were also selected as the target gene to establish a nucleic acid detection method (Qi-Ya, 2003; Seng et al., 2004; Zhang et al., 2010). In this study, we designed and established a rapid detection method of grass carp reovirus type 1 using RPA-based test strips combined with CRISPR Cas13a system. This method has high specificity and sensitivity for grass carp reovirus type 1, and has good practicability in on-site detection. However, in the future, it is necessary to detect the infection of other genotypes of GCRV or additional samples of other species to evaluate their potential application in on-site monitoring of GCRV. For the method proposed in this paper, the theory of establishing the corresponding detection methods of type II and type III is also applicable. In the future, the detection of three genotypes of GCRV in one reaction will be our next research goal.

To sum up, RPA-CRISPR/Cas13a serves as an efficient and precise diagnostic technique to detect GCRV. RPA-CRISPR/Cas13a has significant potential for application in GCRV detection and can be utilized as an effective tool for timely monitoring, prevention, and early transmission control of GCRV.

## Data availability statement

The datasets presented in this study can be found in online repositories. The names of the repository/repositories and accession number(s) can be found in the article/supplementary material.

## Ethics statement

The animal study was approved by Animal experiments in this study were performed according to the Regulations on Administration of Animal Experiments (Ministry of Science and Technology of China, Approval No. 2006–398) and approved by the Animal Ethics Committee of Yangtze University (Jingzhou, Hubei, China). The study was conducted in accordance with the local legislation and institutional requirements.

## Author contributions

HL: Conceptualization, Investigation, Methodology, Software, Writing – original draft, Writing – review & editing. XC: Conceptualization, Data curation, Investigation, Methodology, Software, Writing – original draft, Writing – review & editing. RC: Data curation, Methodology, Software, Conceptualization, Formal analysis, Writing – review & editing. MG: Formal analysis, Investigation, Methodology, Software, Conceptualization, Data curation, Writing – review & editing. MX: Data curation, Formal analysis, Methodology, Software, Conceptualization, Supervision, Writing – review & editing. XW: Data curation, Investigation, Methodology, Software, Formal analysis, Supervision, Writing – review & editing. RY: Data curation, Formal analysis, Methodology, Software, Conceptualization, Supervision, Writing – review & editing. LL: Conceptualization, Data curation, Funding acquisition, Supervision, Formal analysis, Resources, Validation, Visualization, Writing – review & editing. FZ: Conceptualization, Formal analysis, Funding acquisition, Project administration, Resources, Supervision, Validation, Visualization, Writing – original draft, Writing – review & editing, Data curation, Investigation, Methodology.
